# Customized 3D Allogenic Bone Blocks for Mandibular Buccal-Bone Reconstruction Increase Resistance to Tongue-Protrusion Forces: A Finite Element Analysis

**DOI:** 10.3390/jfb16010001

**Published:** 2024-12-24

**Authors:** Sebastian Dominiak, Jennifer Majer, Christoph Bourauel, Ludger Keilig, Tomasz Gedrange

**Affiliations:** 1 Department and Institute of Oral Surgery, Wroclaw Medical University, Krakowska 26 Street, 50-425 Wrocław, Poland; 2 Poliklinik für Kieferorthopädie Dresden, Trefftz-Bau Zellescher Weg 16, 01069 Dresden, Germany; jennifer_helena.majer@tu-dresden.de; 3 Oral Technology, University Hospital Bonn, Welschnonnenstr. 17, 53111 Bonn, Germany; bourauel@uni-bonn.de (C.B.); ludger.keilig@uni-bonn.de (L.K.); 4 Department of Prosthetic Dentistry, Preclinical Education and Materials Science, University Hospital Bonn Welschnonnenstr. 17, 53111 Bonn, Germany

**Keywords:** dehiscence, allograft, bone block, customized bone block, finite elements

## Abstract

Background. The impact of tongue protrusion forces on the formation of malocclusions is well documented in academic literature. In the case of bone dehiscence of the buccal wall in front of the lower frontal teeth, this process may be even more pronounced. Augmentation with 3D customized allogenic bone blocks (CABB) has been proposed as a potential solution for treating such defects. The objective was to assess the impact of bone block adjustment accuracy on the resistance of teeth to protrusion forces at various stages of alveolar bone loss. Methods: A finite element analysis (FEM) was conducted to ascertain whether augmentation with a CABB will result in increased resilience to tongue protrusion forces. Three-dimensional models of the mandible with dehiscenses were created, based on the dehiscences classification and modification proposed in the journal by the authors of regenerative method. The models feature a CABB positioned at three different distances: 0.1 mm, 0.4 mm, and 1.0 mm. The material parameters were as follows: bone (homogenous, isotropic, E = 2 GPa), teeth (E = 20 GPa), periodontal ligament (E = 0.44 MPa), and membrane between bones (E = 3.4 MPa). A tongue protrusion force within the range of 0–5 N was applied to each individual frontal tooth. Results: The use of an CABB has been shown to positively impact the stability of the teeth. The closer the bone block was placed to the alveolar bone, the more stable was the result. The best results were obtained with a ¼ dehiscence and 0.1 mm distance. Conclusions: The protrusive forces produced by the tongue might not be the biggest one, but in a presence of the bone loss they might have serious results. Even shortly after the surgery, CABB has a positive impact on the incisor resilience.

## 1. Introduction

The success of orthodontic treatment is significantly influenced by two key factors: recessions and bone dehiscence. Unfortunately, the prevalence of recessions is quite high, as shown in the meta-analysis conducted by Yadav et al., which found that approximately three-quarters of the studied population experienced some form of recession [1].

Recessions are detected by clinical and radiological examination. In the context of clinical examination, the classifications proposed by Miller and Cairo et al. [2,3] are widely utilized to describe recession as a progressive loss of gingival tissue accompanied by subsequent bone loss in the alveolar ridge. In the case of bone dehiscence, the original classification system proposed by Yang et al. (2015) was later modified by M. Dominiak et al. (2021) [4,5], who provided a more detailed classification of bone loss around teeth. In Class A, the authors incorporated the lingual part. In Class B, authors included both lingual subclasses and subclasses where dehiscence reaches the apical foramen, coexisting periapical lesions, and coexisting fenestration or unilateral dehiscence. In Class C, a distinction was drawn between the levels of dehiscence observed on the two sides. In addition, a completely new Class X has been introduced to describe instances where dehiscence is not visible upon clinical examination.

The classification system proposed by Geiser [6] is similar to the one presented here. Glickman has divided recessions into two categories: visible and invisible. Visible recession involves both alveolar bone loss and adjacent gingival loss. In contrast, an invisible recession is defined as bone loss on the labial and/or lingual surface of the dental ridge. Such defects are referred to as dehiscence, which is defined as a V-shaped defect located on the bone margin toward the apex. They can be observed on the buccal or lingual side of a tooth. Another example of bony defects in sagittal direction are fenestrations, defined as window-like openings of the alveolar bone that involve the root apex of the associated tooth.

It is important to consider the bone conditions of the alveolar process when planning orthodontic treatment, as the risk of developing recession is relatively common. In some cases, the tooth may move outside the alveolar envelope, particularly if the process is narrow or there is a bone dehiscence [7]. It is therefore crucial to accurately diagnose the presence and extent of recession or dehiscence when determining the course of orthodontic treatment.

It was found that the probability of recession development is greater when the angle of the labial bone width is below 16 degrees. The anticipated tooth movement is also a significant factor. In the case of protrusions, it was found that multiple recessions were more prevalent, while retrusions were single and of greater height. It is crucial to consider the type of malocclusion and the musculoskeletal factors affecting the condition of the surrounding bone and soft tissues of the alveolar process [8].

Various types of bone graft substitutes have been used to avoid these complications and to achieve a wider alveolar ridge. Most of them have been performed according to the same pattern, i.e., Guided Bone Regeneration (GBR), where bone graft substitutes were used in the form of granules and covered with pinned collagen membranes. The main advantages of this technique are easy-to-buy materials, speed, and uncomplicated procedure. However, it is important to address its potential drawbacks, such as low predictability of the procedure due to poor width growth after reconstruction, displacement of bone material during the healing period due to gravity and muscle tension, and most importantly, the quality of the reconstructed dental arch. In many cases, it was a conglomerate of bone substitute and newly formed bone that lacked the characteristics of the vital bone. It created areas of higher density, which allowed orthodontic movements and were starting points of root resorption. Therefore, it becomes clear why there is no place for the regeneration of dental arches with all teeth preserved, especially when orthodontic treatment is planned [9].

To overcome these difficulties, M. Dominiak, T. Gedrange et al. have developed a surgical procedure using a customized 3D-allograft to restore alveolar bone prior to orthodontic treatment or in cases with a poor treatment plan. Enlargement of the alveolar bone envelope allows completion of orthodontic procedures without major complications. In this CT-based technique, we create a custom-milled bone fragment that protects, strengthens, and rebuilds the labial wall of the mandibular alveolar ridge to provide additional space for the planned placement of teeth [9].

The most important part of this procedure is the preparation of the block. Bone implant consists only of the spongy bone, which on one hand allows faster revascularization, but on the other increases fragility, so the surface of the bone block may not be as ideal as planned. Typically, the gap between the allograft and the residual bone does not exceed 0.4 mm. Therefore, revascularization should proceed smoothly. It is also important to ensure the accuracy of the bone millers. The accuracy of the milling process varies between different machines. It is therefore essential to work with the best possible equipment. The accuracy of the milling process of the blocks used in this process is set at 5 µm. However, the pore size of the cancellous bone is up to 500 µm [10]. As a result, it is possible that, despite the accuracy of the process, imperfections may be detected on the surface of the bone block. Furthermore, the accuracy of converting CT scans to planning models and the possibility of bone chipping during the surgical procedure are also factors to consider.

The main objective of this study was to evaluate whether the accuracy of the bone block adjustment affects the teeth’ resistance to protrusion forces at different stages of alveolar bone loss, even shortly after surgery. An additional objective was to determine how accuracy of the bone block fit will affect healing time and quality.

## 2. Materials and Methods

The finite element method (FEM) is a numerical technique for investigating biomechanical properties without costly and traumatic procedures on patients and/or animals The technique was first introduced in the field of dentistry about 50 years ago [11], and can be employed to demonstrate the presence of stresses and strains within the tissues.

**Figure 1:**
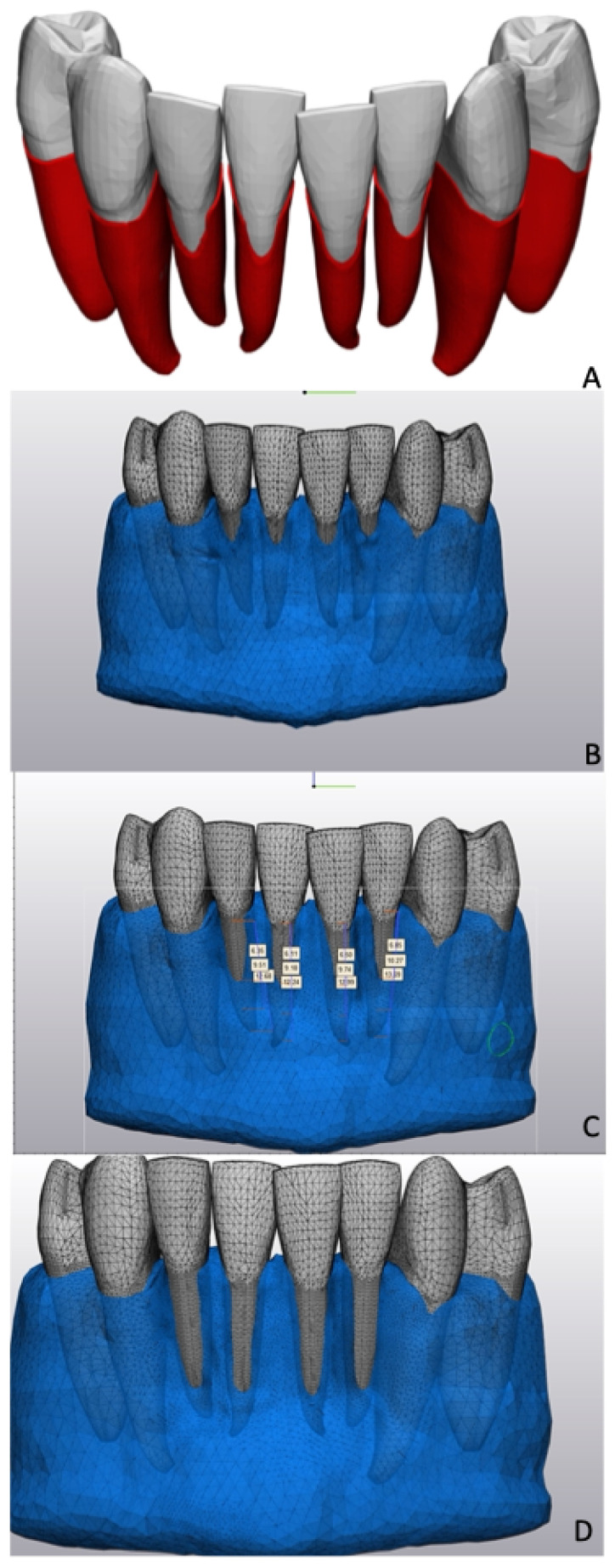
Initial models with dehiscence. (**A**) Model with visible PDL and invisible bone at ¼ dehiscence level; (**B**) initial model at ¼ dehiscence level; (**C**) initial model at 1/2 dehiscence level; (**D**) initial model at 3/4 dehiscence level.

All models have been designed in Finite Element (FE) software Materialise 3-matic version 15.0 (Materialise N.V., Leuven, Belgium). A commercially available idealized surface model of the lower jaw, comprising teeth with roots and gum (Viewpoint Data Labs [now Digimation Inc., Lake Mary, FL, USA]), was employed as a preliminary template for the generation of FE models. The objective of this study was to examine the impact of bone resorption on the anterior teeth (canine and incisors). To this end, all molars and the second premolar were excluded from the base model. The first premolars were left in the model, to ensure that the immediate anatomical neighborhood of all the examined teeth, including the canines, showed a realistic clinical surrounding. Since this model represented an ideal dental arch with no malocclusions, the remaining teeth were repositioned to reflect the crown positions found in a clinical case. The positioning of the teeth in the models has been designed to reproduce the authentic crowding that the clinician may observe in the practice. The resulting teeth with periodontal dental ligaments (PDLs) positions without the bone part of the anterior mandible are shown in Figure 1A. From this baseline situation, we created 3 models of the jaw bone with 3 levels of vestibular bone dehiscence, as described by Dominiak et al., with ¼, ½, and ¾ of the roots exposed (Figure 1B–D). We only created dehiscence in front of the incisors, as observed in the clinical studies.

The original base model lacked periodontal ligament (PDL), so we created an idealized PDL with a constant thickness of 0.2 mm [12,13,14] around that part of the root within the bone. A separate allograft bone block (ZimVie^®^, Germany GmbH, Munich, Germany) was prepared for each of the above models. The objective of the bone substitute was to recreate the horseshoe-like shape of the standard mandible, which is anatomically correct, and to ensure that it did not overlap with the mentalis muscle in the vertical dimension. Therefore, the models did not have average bone-block thickness. To modify the distance between the mandible and the bone blocks, three different trim distances were applied to the existing bone/tooth surface: 0.1 mm, 0.4 mm, and 1.0 mm. This was done to mimic imperfections or poorly milled grafts. The bone-block design procedure was described in more detail in the previous article [9]. A soft-tissue membrane was placed between the teeth and the allogenic bone, to represent the clinically applied Advanced Platelet Rich Fibrin (A-PRF) membrane. In a final step, titanium screws were inserted to mimic the stabilization of a bone block in a clinical application. As no CAD data for these screws were available, two screws (titanium alloy SD screws for GBR system, length 6 mm and 8 mm, both of 1.2 mm diameter, Surgident Co., Ltd., Daegu, Republic of Korea) were scanned in an in-house µCT scanner (Skyscan 1174, Bruker, Kontich, Belgium) and reconstructed using Mimics 23 (Materialise N.V., Leuven, Belgium), and were placed in three different places for block fixation. In total, we created nine different models: three different degrees of vestibular compact-bone dehiscence, each with three different positions for the allogenic bone blocks.

**Figure 2:**
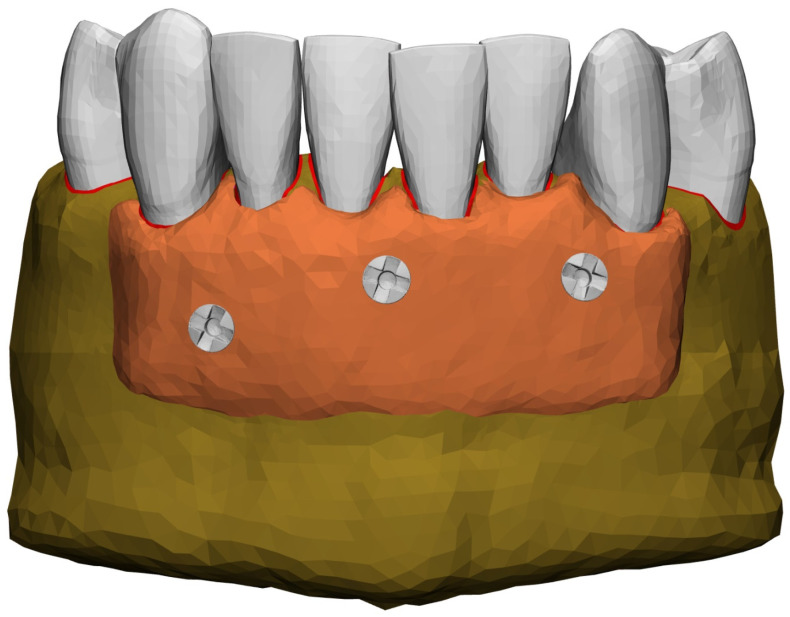
Finished model with stage 1 dehiscence and bone block placed 0.1 mm from the native bone.

Figure 2 shows the final model. Degenerated 8-node hexahedral elements were used for the soft tissues (PDL and membrane), while 4-node tetrahedral elements were used in the remaining parts of the model (bone, teeth and screws). Element sizes have been selected according to the required geometric details. For the PDL, the maximum element edge size was set to 0.1 mm in the thickness direction. Element sizes in the crowns of the teeth were 0.8 mm, increasing to up to 2.0 mm in the outer regions of the bone. The final models consisted of 108,000–113,000 nodes and 472,000–501,000 elements.

Bone, teeth and the autogenous bone block were all considered as homogeneous material, with no further differentiation between cortical and spongy bone or between dentin and enamel. Table 1 shows the material parameters of all the materials involved. Special consideration was given to the material description of the soft tissues: the models were created to mimic the natural situation approximately one week after surgery, i.e., there was no more bleeding and the process of osseointegration was at an early stage. The membrane was still intact and/or the connective tissue showed no signs of calcification.

Protrusive forces are applied separately to the incisal edge of each crown (central and lateral incisors and canines) with a force of up to 5 N, mimicking anatomical muscle forces). All simulations were prepared and performed in the FE software package MSC Marc/Mentat, version 2020 (Hexagon AB, Stockholm, Sweden).

## 3. Results

The following parameters were investigated in our simulations: tooth displacement, PDL, and membrane strains. To properly show detailed results, they were divided into several groups and subgroups, according to the amount of bone loss in front of the teeth and the distance between the patient’s bone and the allogenic block.

### 3.1. Tooth Displacement

**Figure 3:**
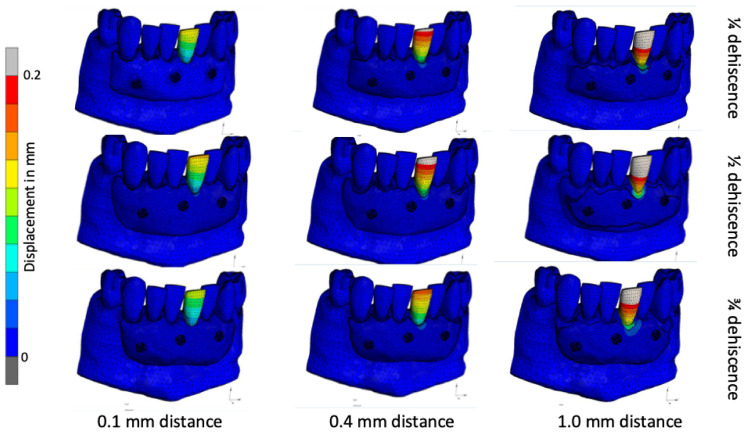
Tooth 31 displacement with the presence of ¼, ½ and ¾ root exposure at 0.1–1 mm spacing.

As an example of the calculated displacements in the teeth, Figure 3 shows the calculated displacement maps for loading tooth 31 in different model configurations (¼, ½ and ¾ residual bone, with bone plates at 0.1 mm, 0.4 mm and 1.0 mm spacing). The displacement of the tooth due to the protrusion forces generated from the lingual side usually deviates from the natural situation, i.e., the greater the dehiscence, the greater the displacement of the tooth. The displacement of the crown of tooth 31 was found to be higher when a bone plate distance of 1.0 mm was used, regardless of the level of root exposure. For example, at ¾ resorption, the crown moved 0.12 mm, 0.18 mm and 0.27 mm for bone block distances of 0.1 mm, 0.4 mm and 1.0 mm, respectively. Furthermore, the simulation indicated that the displacement was reaching the bone block. The displacement on the allograft reached higher values and a larger area, while the dehiscence was more severe. The models with the closest fit to the milled block also showed the smallest observed displacement on the bone block. For instance, in the case of a bone block located on a dental arch with dehiscence extending to ¾ of the root length, the displacement was observed to be 0.001–0.035 mm for distances of 0.1 mm and 1.0 mm, respectively.

**Figure 4:**
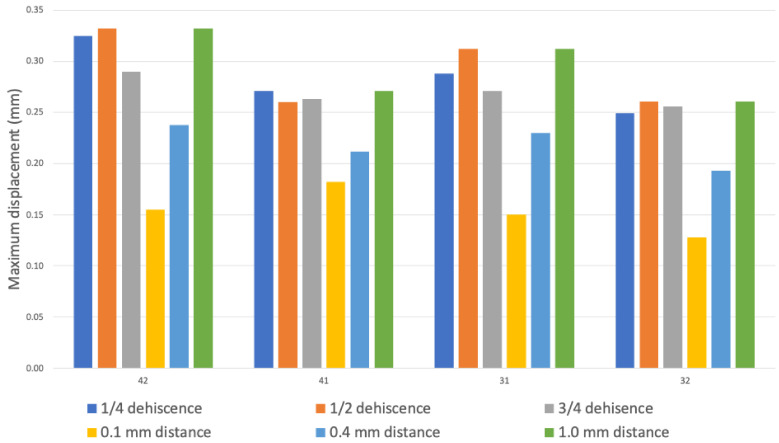
The bar graphs illustrate the maximum displacement in relation to the level of dehiscence and the distance to the bone block on each tooth. The blue, orange, and gray bars represent the maximum displacement for different stages of bone dehiscence alone. Yellow, light blue and green are representing maximum displacement for different distances between the maternal bone and the bone block.

Figure 4 presents the maximum displacement determined for all teeth in different model configurations, illustrated as a bar graph. Teeth 43 and 33 were excluded because they were not in the range of the designed bone dehiscence. The greatest displacement was observed on protruding teeth 31 and 42, reaching around 0.3 mm, but the highest score was not achieved with ¾ dehiscence, but rather with ½ dehiscence. Unsurprisingly, the highest value of displacement was observed at the longest distance between the bone elements, i.e., at 1.0 mm distance (green bar), and around each tooth it exceeded 0.25 mm. In contrast, models in which the bone block was positioned 0.1 mm further away from the dental arch exhibited values that were approximately 0.1 mm lower.

### 3.2. Strains in PDL

The PDL absorbs the force generated by the lingual pressure. The more force applied to the tooth, the greater the strain. The strains present in the PDL when force is applied to the teeth have the highest values around the cementoenamel junction and the apex of the tooth. They are located on different sides of the PDL—the more coronal the strains on the lingual side, the more the apical strains reach higher values on the labial side. This is caused by the tipping movement of the tooth, where the vestibular movement of the crown results in a lingual movement of the apex of the root.

**Figure 5:**
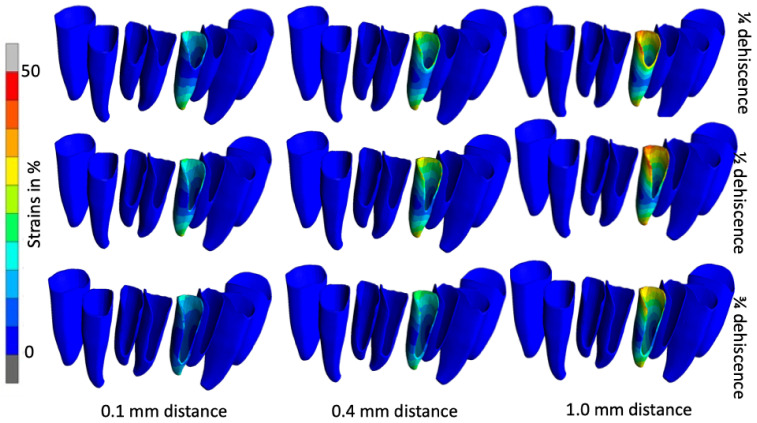
Strains in PDL 31 with the presence of ¼, ½ and ¾ root exposure at the distances of 0.1–1 mm.

The discrepancy between the stresses accumulated in the PDL is clearly visible in Figure 5. The dehiscence is relatively small. However, if the block does not fit exactly on the bone, (1.0 mm distance), the forces reach more than 50%, whereas if the distance is only 0.1 mm, they reach only 30%.

**Figure 6:**
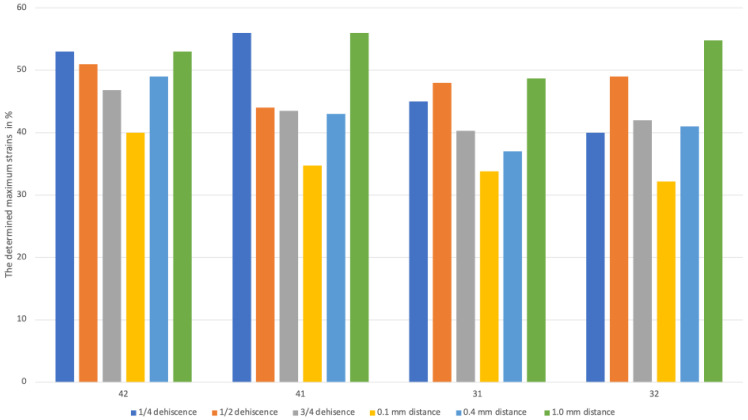
The bar graphs illustrate the maximum strains in the PDL for all models and all force applications. The blue, orange, and gray bars represent the maximum strains for different stages of bone dehiscence. The yellow, light blue, and green bars represent the maximum strains for different distances between the maternal bone and the bone block.

Despite the enlargement of the dehiscence to half of the root, the visible strains were slightly lower than in the previous example. The 0.1 mm width of the membrane ensures lower strain values in the coronal and apical regions, which are approximately 20–25%. In contrast, a 1.0 mm wide membrane resulted in 50% strain values at the PDL border, with an average of 40% strain values observed. Figure 5 shows the distribution of the strains in the PDL when loading the left lateral incisor (tooth 31). Figure 6 shows the determined maximum strains in the PDL for all the models and force applications. The tendency to lower the tension as the dehiscence increases is also achieved when the root is exposed to ¾ of its length. However, the forces are relatively small. In the case of 0.1 mm, they reached only 15%, and in the case of a 1 mm gap between the autogenous bone and the graft, they fluctuated around 30%. With a gap of 0.4 mm, the strain level is still relatively low in all cases. It oscillates around half the scale, i.e., around 25%. However, with a quarter root, the exposure forces are slightly higher at the edges—in the region of the apex and around the CEJ on the lingual side. With the widest area between the grafted bone and the autogenous bone, the level of strain in the PDL is significantly higher than in the previous examples. The block placed in this way does not stabilize the teeth after surgery, and physiological movements of the tongue will worsen the healing process. In the case of ¼ dehiscence, a rather large area reached a maximum of 50%, especially in the region of the top of the PDL.

### 3.3. Position of the Center of Rotation

**Figure 7:**
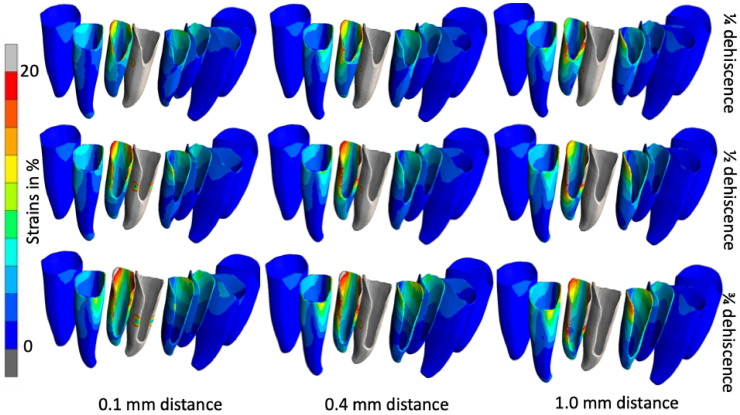
The location of the center of rotation on various teeth with different degrees of dehiscence and different membrane widths.

The strains in the PDL are induced by the relative movement of the tooth in its bony socket, and this movement consists mainly of a tipping movement of the crown to the labial side. Therefore, the distribution of strains in the PDL can be used to visualize the movement pattern of the loaded tooth. The region with the lowest strain in the PDL corresponds to the center of rotation (CoR). In the case of forces applied to the crown apex from the lingual side, the center of rotation passes more labially, and increases in line with a reduction in the membrane thickness. The CoR moves more apically and labially as the degree of dehiscence increases. Figure 7 illustrates the correlation between the stages of dehiscence, the width of the membrane, and CoR.

### 3.4. Strains in Membrane

**Figure 8:**
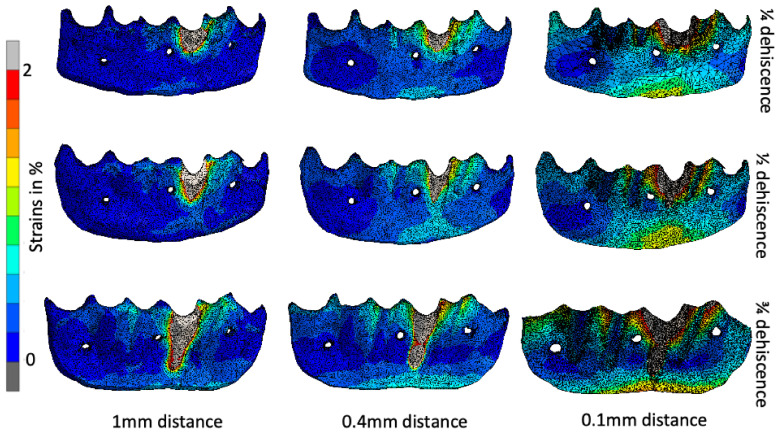
Strain distribution in the presence of ¼, ½ and ¾ root exposure at the 0.1–1 mm distance.

Figure 8 shows the strains in the membrane resulting from the load applied to tooth 31 in two selected models. These strains are caused by the labial displacement of the teeth, and are directly proportional to the severity of the dehiscence. The strains manifest along the inferior border of the membrane. In our simulations, we found that the strains decreased with increasing membrane thickness. With more fibrin fibers, the membrane between the block and the patient’s own bone can better distribute the strains. The strains observed represented a relatively small percentage of the total, not exceeding 10%. To enhance clarity and presentation, the scale has been reduced to 2%.

In the case of a 0.1 mm wide membrane and ¼ dehiscence, strains are located in two-thirds of the whole membrane and exceed 2% around the tooth cervix. In the case of a 1 mm wide membrane, strains are located around the dehiscence area. In the case of ½ dehiscence, strains tend to manifest similarly to those observed in the ¼ dehiscence case. However, they also reach higher levels around the root surface and in the mental area. In membranes measuring 0.1 mm, 0.4 mm, and 1 mm in width, forces accumulate in the exposed root area, reaching levels exceeding 2%. In the case of thinner membranes, inferior border forces reach approximately half of the scale. In thicker membranes, they reach approximately 0.5%. The growth of strains in the ¾ stage of dehiscence is directly proportional to the enlargement of the dehiscence. In the case of the thinnest membrane, almost the entire area reaches approximately 1%, while in the case of the thickest membrane, strains concentrate generally around the dehiscence, reaching approximately 0.4%.

The pattern of strain locations is quite different at each level of bone resorption, despite the same membrane thickness. In the case of ¾ dehiscence, there is an area in the middle with no tension, due to the balancing forces from the lower and upper boundaries. At both ends, the value of the force reaches the upper limit of the scale. In the case of ¼ dehiscence, the forces are distributed more evenly, with a high concentration around the tooth neck in both cases. The ½ dehiscence appears to represent a transitional phase in the strain distribution.

The situation in the 0.4 mm thick membrane represents a balance between absorbing the thick membrane and the thin one, which distributes forces on the entire membrane. The strains are more or less arranged along the long axis of the tooth. In the rest of the membrane, there are almost no strains. As anticipated, the strains exceeded 2% at the root surfaces.

Due to the higher number of fibers in the 1 mm membrane, in all cases the strains were concentrated almost exactly around the dehiscence, leaving the rest of the membrane free of strains. If 1 mm thickness does not disturb revascularization, it will work well as a strain absorbent. However, due to the fact that a greater amount of tissue has to be reorganized to the newly formed bone, this situation is not desired.

**Figure 9:**
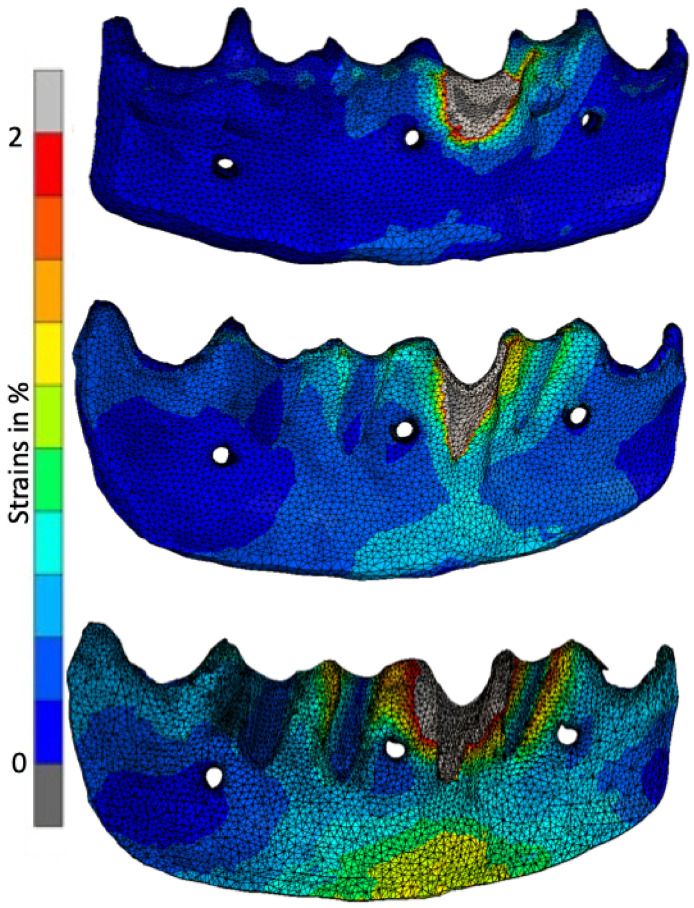
The pattern of stress accumulation around the screws that is reduced, regardless of the dehiscence stage or membrane width.

It is also worth noting that the areas where the screws were placed are free of stress. The screws are the cause of the low strains around them. Because the screws are rigid and anchored in the jaw bone, and the membrane is soft and elastic, the bone block wiggles on the top of the membrane. The bone block is tipping, with the screws serving as a hinge. Therefore, the bone block is tipping around the screw heads, bringing the bottom edge closer to the bone in the apical region. This is also the reason why strains are visible at both the top and bottom edges of the membrane, as shown in Figure 9, at various dehiscence levels and membrane widths. At the top, the block has been pushed away, causing pressure (compression) in the membrane between the mobile tooth and the bone plate, and tension in the membrane between the immobile neighboring teeth and the moved bone block. At the lower edge, the membrane is again compressed between the tipping bone plate and the fixed bone.

## 4. Discussion

The issue of dehiscence around anterior teeth represents a significant challenge in contemporary dentistry. While it has been observed to worsen over time, it was not as prevalent a century ago. An examination of epidemiological studies from recent times [18] and approximately 75 years ago [19] reveals a notable shift in the mean appearance of Class II malocclusion. However, the Class II angle is not synonymous with thin gingival biotype, but as it is the most common malocclusion which is treated in orthodontic departments [20] and orthodontic treatment, it is strictly connected with the appearance of gingival recessions [21]. These morphological changes might be explained by bad habits, processed food, and lack of vitamin D_3_ due to changed lifestyle, among many others [22,23,24].

Pre-orthodontic examination based on CT scans should be considered as a new “gold standard”. Thanks to its 3D visualization of hard tissue, the width of bone which surrounds the teeth can be checked. With just a sufficient distance between bone borders from the tooth apex, a lower risk of bone defects after orthodontic treatment can be guaranteed [25].

Orthodontic treatment can cause gingival recession and bone dehiscence. The most common factor is teeth moving through the dental arch labially [26]. In contrast, the findings of M. Dominiak indicate that even when teeth are tilted lingually, with the center of rotation situated in the region of the alveolar margin, bone resorption occurs [8].

Regenerative procedures in dental surgery are a well-known topic. They can be divided according to their material of origin: whether they are obtained from the patients, or others from the same species, derived from animals, or corals or synthetic materials [27]. They can also be divided by the different properties of the material. Grafts can be manufactured in a variety of forms, including granules covered with membrane or metal scaffolds, plates, or bone blocks. Although the methods differ, their goal is the same: to create sufficient bone volume for the implant [28,29]. Some other scientists also performed regenerative surgery in alveolar bone defects using CAD/CAM-based procedures, with positive results. However, none of them used their techniques to enlarge the bony envelope around teeth [28,29,30,31].

Only a few studies have reported augmentation in the dentulous ridge. These include Brugnami et al., who performed guided bone regeneration (GBR) with corticotomy to allow root movement through the labial wall [32]. However, the use of soft membranes can be the cause of bone particle migration due to muscle tension. This type of movement in itself may prolong healing time, but may also be the reason for the creation of bone graft conglomerates with ingrown soft tissue fibers, without any evidence of vital bone [33].

A similar conclusion was reached by Farah Asa’ad et al., who investigated the use of 3D scaffolds in regenerative surgery related to implant therapy, as well as their potential application in periodontal therapy [34]. They discussed the potential use of 3D printed scaffolds made of different materials. However, osteoinductive properties cannot be achieved by using a biomaterial from a different species [27].

The findings indicated that an increased distance between the maternal bone and the graft resulted in a lack of stability for the block on the bone surface, as well as the formation of hollow areas. Blood will accumulate in these (empty) areas after the procedure. If a hematoma is organized, it will delay the revascularization process by having to break down on its own. The same situation occurs in tooth sockets after extraction or during healing after bone fractures. Another objective reason for poor healing is spontaneous tooth migration. It can occur especially when there is dehiscence on the labial side along with bone loss in the interproximal spaces, which may occur in periodontal disease. We can also add a large concavity of the alveolar part of the mandible with many curvatures, which makes it difficult to achieve a close distance.

The available literature on FEM simulation of tooth loading with anatomical forces is scarce. It focuses more on orthodontic movements or implantology. However, if we take a larger scale, we will observe similar conclusions. For instance, Sioustis et al. conducted a comparative analysis of bodily movement during orthodontic treatment in normal and periodontally compromised dentition. They applied forces within the range of 0.8–1 N in the bodily direction to simulate orthodontic treatment with braces on teeth affected by periodontal disease. They found that tooth morphology and PDL thickness should be taken into account. The thinnest PDL correlated with the greatest bone displacement [35]. A comparison of their results with our own assessments reveals a clear pattern. Periodontally compromised teeth are much less able to absorb forces, so already deformed alveolar bone is exposed to greater stresses and strains, which can lead to further bone resorption and enlargement of the dehiscence.

Baghdadi et al. presented their results in a similar tone [15]. They were able to assess that teeth with reduced periodontium should be treated with lower forces, due to the greater load on the periodontium with increased tooth movement. While this study has a fixed orthodontic treatment in common with other studies, they agree that the forces should be lower, due to the lack of bone support, which is strictly related to the volume of PDL around the teeth. It is only its ability for stress and strain absorption and displacement that allows for a predictable and safe treatment.

Amid et al. evaluated the influence of splinting anterior teeth in the mandible with compromised periodontium. They used fiber-reinforced composite for their purpose. They stated that splinting decreased the magnitude of stresses and strains in the area of incisors. Comparing it our results, we obtained similar data, despite using lesser forces (in our study, up to 5 N, in theirs, 100–200 N). As the bone block was placed closer to the teeth and bone, it gained splinting ability, and the strains shown in our study were lesser in cases of a better block fit [36].

The results confirm that the regeneration process itself is not sufficient to stabilize the frontal teeth. Its precise methodology and tight fit only allow for a faster healing time, due to the reduced mobility of the bone substitute. A stable individualized bone block has a great influence on the stability of the teeth almost from the time of surgery, especially when the distance between the bones varies by 0.1 mm. Important information for block design is that the desired stiffness depends on the block fit and block width. Placing a membrane under the bone block has a positive effect, as it also absorbs the horizontal forces caused by the patient’s tongue movement. However, if it is too thick, i.e., the distance between the tooth and the bone substitute reaches 1.0 mm, even if the dehiscence is in its early stages, the displacement transferred to the block will also be observed.

The results of this and other studies should be considered in light of the potential risks associated with gingival recession, loss of bone support, and, ultimately, tooth loss due to inadequate diagnostic procedures. These should be based on CBCT scans prior to orthodontic treatment, which should make it faster, more predictable, and safer. However, if the bone defects are already present, the method of choice should be bone regeneration, which ensures stability and rapid healing time. One of these surgeries should be performed with a customized 3D allogenic bone block. The results of this method have been very encouraging in a follow-up period of almost 5 years, and will be described in future studies. This method can be used to reduce complications during orthodontic treatment in compromised cases, prolong the natural dentition, and delay the placement of implants in a frontal region that is difficult with respect to prosthetic work.

## 5. Limitations

The FEM provides a cost-effective and straightforward way to assess the clinical characteristics of scenarios encountered in practice, eliminating the need for complex prospective clinical studies. However, more advanced simulations require many hours of design time, and sometimes fail to show the intricate connections and properties of the tissues. The FEM is primarily designed to address a specific, complex problem. However, our research demonstrates that investigating individual problems within the FEM framework can also yield valuable insights.

Unfortunately, we lacked designed soft tissue surrounding the bones and teeth in the nine FE models we created. To be more precise, the gingiva (the mucous membrane of the alveolar part of the mandible) and the muscles placed around the designed area affect both teeth mobility and healing after grafting procedures. However, in a simple way, possible strains and their effect on tooth mobility and stability of the grafted material are presented. The forces used in the simulations are shown for the individual tooth (we focused on the incisors), but using all of the information collected could help visualize the problem of excessive protrusion forces and their influence on dental arches.

In the presented models, we used the still-existing membrane without signs of its resorption; i.e., models represent the clinical situation immediately after the surgical procedure. Despite the early stage of healing, the models demonstrate the influence of tooth stability, particularly in the 0.1 mm distance between the bone block and the dental arch. As the healing process progresses, stability is expected to increase.

## 6. Conclusions

Dentition with compromised surrounding bone structure might be easily affected by the natural forces created by the muscles. Even shortly after surgical procedure, stable bone substitute will have a positive impact on the resilience of the frontal teeth to protrusion forces. However, only the tight fit of the bone block, which does not exceed 0.4 mm, can increase the resistance to forces. It allows faster osteointegration and improvement in the potential of regeneration, as well.

## Figures and Tables

**Table 1:** Material parameters used in the simulations.

Material	Young’s Modulus	Poisson’s Ratio	Source
Bone (not differentiated)	2000 MPa	0.30	Baghdadi et al., 2019 [15]
Teeth (non-differentiated)	20,000 MPa	0.30	Baghdadi et al., 2019 [15]
Bone block			Same as bone
Screws (titanium grade V)	110,000 MPa	0.28	Material data sheet from the manufacturer
Periodontal ligament	0.44 MPa	0.30	Yoshida et al., 2001 [16]
Membrane	3.4 MPa	0.45	Wada et al., 2006 [17]

## Data Availability

The original contributions presented in the study are included in the article, further inquiries can be directed to the corresponding author.
